# 从美国住院医生培训看中国住院医师规范化培训的挑战和方向

**DOI:** 10.3779/j.issn.1009-3419.2016.06.03

**Published:** 2016-06-20

**Authors:** 永 崔, 天佑 王

**Affiliations:** 100050 北京，首都医科大学附属北京友谊医院胸外科 Department of Thoracic Surgery, Beijing Friendship Hospital, Capital Medical University, Beijing 100050, China

**Keywords:** 规范化培训, 住院医师, Standardized training, Resident doctor

## Abstract

住院医师规范化培训在我国已经全面启动并逐步铺开。由于住院医师规范化培训是临床医学毕业生成长为合格临床医师的重要途径，是保证临床医师均质化、提高医疗服务质量和水平的治本之策，所以此项工作受到各方瞩目。美国的住院医生培训已有近百年的历史，形成较为系统的模式也已有近50年，是西方医学教育的典型代表和成功模式。本文旨在通过比较中美两国在培训的目标、计划、管理机构、考核和薪酬等方面的差异，对我国规培制度的现行的制度安排和发展方向进行讨论。

## Abstract (en)

Resident standardization training has been started and spreaded out gradually in China. Resident standardization training is crucial to ensure the clinician homogenization, improve medical service quality and level of medical treatment and health care, so it received much attention from all sides. Residency training in American has a history of nearly a century. Systematic model of residency training in the United States had been established for nearly 50 years, and it is a typical representative and successful example of the western medical education. The purpose of this paper is to discuss the institutional arrangements and development direction of the resident standardization training in China, based on comparison of the two residency training system between the two countries on target, schedule, management institution, evaluation and remuneration.

## 1 背景

1993年，卫生部印发《关于实施临床住院医师规范化培训试行办法的通知》，此后各地逐步开展不同规模、不同水平的住院医师规范化培训的前期探索。2013年底，国家卫生计生委联合教育、财政、人社等6部门制定出台了《关于建立住院医师规范化培训制度的指导意见》，2014年中国国家卫计委正式公布《关于建立住院医师规范化培训制度的指导意见》和第一批住院医师规范化培训基地共371家，2015年在全国全面启动住院医师规范化培训工作，目前，住培招收工作已在全国31个省（区、市）铺开，2015年全面启动的制度预期目标顺利实现。美国的医学教育和住院医培训是西方医学教育的典型代表，是比较成功的教育模式。其住院医师规范化培养制度始于20世纪20年代，并逐渐成为美国医学教育过程中的重要组成部分。自1917年开始，以住院医师培训的评价和认可为目的的各专科委员会相继成立，至1972年美国毕业后医学教育联络委员会（Accreditation Council for Graduate Medical Education, ACGME）成立，美国形成了完整的毕业后医学教育评价与认可体系^[1]^。中国目前在很多方面也在积极学习美国体系。国外的医学教育实践充分证明，住院医师规范化培训是临床医学毕业生成长为合格临床医师的必由之路，是保证临床医师均质化、提高医疗服务质量和水平的治本之策。在讨论规范化培养之前，必须强调一点，无论何种培养体系，都会有医学大师产生，规范化培养只能保证达到专科医生的最低标准。本文旨在通过比较中美两国在规培制度设计上的差异，对中国的规培制度的完善和发展进行讨论。

## 2 美国住院医生培训概况

在美国，申请住院医师培训时必须具备以下条件：①4年的理工科大学本科的学习，完成相应课程学习并且成绩优异，通过美国医学院入学考试（The Medical College Admission Test, MCAT）考试，并具备研究经验、志愿者经历和领导能力；②4年的医学院学习。全美每年医学院招生总规模在16, 000名左右。前两年为基础学习，结束时需参加全美医师资格考试的第一步考试（step 1），此考试对阅读、判断、思维和写作能力都有很高的要求，通过测试后方可进入高年级的学习。后两年为临床教学，毕业前进行第二步考试（step 2）。通过前两步考试后，可以报名申请住院医师培训，然后医院面试决定是否给予职位（match program）。全美有1, 700所医院接受住院医师培训。每一名毕业生平均要向26家医院申请，某些专业可能需要提出更多的申请，如外科通常需要向100家以上的医院提出申请，申请成功的概率在外科一般低于1%。对首选专业，平均要接受10家-20家医院的面试。毕业生能在全国著名医院竞争到多少住院医师席位是衡量该学校医学教育质量的重要指标。住院医师训练结束时，进行医师资格考试的最后一步（step 3），通过后便具备了基本的行医的资格。每年5月毕业后医学教育联合委员会召集会议，决定下年度全国住院医师培养计划，汇编成住院医师培养计划指南（Directory of Residency Training Programs），经由医学教育委员会认可后向全美以及国外医学院毕业生提供训练职位。

## 3 培训的目标和计划

美国住院医师培训的目标是“使住院医生毕业后能够成为主治医师，工作独当一面，在激烈的市场竞争中求得生存”。在美国，几乎所有医学毕业生都要接受至少3年的住院医师培训，某些专业长达8年。内科要求为3年，皮肤科要求1年内科加3年专科，而胸外科则要求5年普外科加3年专科。我们以普外住院医师为例介绍其5年的培训流程。第一年为实习医师（intern），入院第一周学习临床工作注意事项，熟悉医院情况。通过第一部分基本生命支持（Basic Life Support, BLS）培训，即心脏按摩、人工呼吸、气管插管等技巧和第二部分高级生命支持（Advanced Cardiac Life Support, ACLS）培训，即抢救患者的标准化流程。第二周开始临床培训，由总住院医师领导。实习医师主要任务是管理住院患者，值班时在病房，不值班的时候要去手术室，每周有1天-2天要去门诊，门诊患者看完后必须经过主治医师过目修改。第一年住院医师结束，通过Step 3美国医师执照考试，获得住院医生许可（Residency Permit），此时尚不能独立处理患者，在多数的州，还需要经过2年-3年的训练才可以获得普通许可（Regular License）。二年级住院医师的培训较第一年难度有所增加，开始到重症加强护理病房（intensive care unit, ICU）及一些专科病房（小儿外科、创伤外科、移植、整形烧伤、胸外科等）进行轮转。三年级住院医师要主刀中等以上手术如胆囊切除、乳房切除等，并开始做急诊会诊工作。急诊患者首先由急诊室医生完成基本检查和诊断，然后交由三年住院医师决定是进手术室手术还是收入病房。此外要管理会诊的患者，但不具体负责外科住院患者床位。四年级住院医师开始做住院总医师（chief resident）工作，包括做四个月的创伤外科住院总医师、四个月的血管外科住院总医师，四个月小儿外科，或普外科，亦或胸外科的住院总医师。第五年做普外科住院总医师，包括排值班、排手术、查房、主刀较大的外科手术，任何手术只要住院总医师想做均可参加，另外还要管理低年住院医师、负责自己的患者、选择病例作教学材料、查房、负责死亡病例和并发症的讨论等工作。由此可以看出，普外科住院医师培训计划要在5年内将一个医学院校毕业生培养成为能够独立工作的普外科医师，使其可以基本胜任普外科的临床工作，能够独立完成普外科的常规手术。但多数住院医生会进一步参加fellowship（进修医生培训）的训练。

中国国家卫生计生委、教育部、国家中医药管理局于2014年11月联合召开“医教协同深化临床医学人才培养改革工作推动会”，对2015年住院医师规范化培训（简称规培）工作的全面开展提出指导性意见，明确提出要在5年内，也就是到2020年在中国全面实行规培。这一制度在全国的推广具有非常重要的现实意义，全国上下都对此制度寄予厚望，是2009年新医改以来少有的亮点之一。近平总书记曾经讲过，要“推动医疗服务均等化，缓解大医院始终处于‘战时状态’”。从这个角度理解规培的目标，应该是建立全国统一的住院医师培训标准，实现全国不同地区、不同级别的医院住院医师的标准化。执业医生的认证和行业管理是医生从单位人变为社会人的前提，而住院医师规范化培训是实行执业医生的行业管理和认证的基础。因此，规培制度有望促进公立医院的人事制度改革，医生由单位人变为社会人，为实现医生的自由职业铺平道路。卫计委提出的规培的具体目标是“为各级医疗机构培养具有良好的职业道德、扎实的医学理论知识和临床诊疗技能，能独立诊治常见病、多发病的合格医师”。主要包括四方面的内容:政治思想、职业道德、专业能力、教学与科研^[2]^。三年规培结束并通过考核取得所需的证书之后，便具备了独立行医的资格。中国参加住院医师规培的医师主要包括三类，有工作单位或没有工作单位的医学院校毕业生和在读医学硕士研究生，绝大多数为没有临床工作经验的本科毕业生。与美国的同行相比，他们只经历了5年大学学校教育（美国是8年），其年龄小、社会阅历少、所经历的竞争的强度也较低，换言之，中国的住院医师培训门槛较低。在时间安排上，中国对各学科的安排都是3年，没有根据不同的专业要求进行细分，而且在很多专业中低于美国的年限要求，更没有亚专科轮转的安排。另外，不同于美国按年度阶梯对住院医有明确的进阶要求，中国的规培计划是3年不同科室的平行大轮转，这是目前中国规培的最大问题之一，应借鉴美国的成功经验，制定具体的年度培训计划。我们必须认识到，中美两国的住院医生虽然都名为住院医生，但实际接受的训练和临床水平完全不在同一级别。美国多数医院的专业职称仅有住院医生和主治医生，与中国的职称体系大不相同。如果做一比较，完成美国的住院医师培训的医生水平大底应相当于中国高年主治医师的水平。在赋予行医资格和相应权利时，必须考虑这一情况。

在完成住院医师培训之后，如果准备从事专科工作，美国要求在完成3年的住院医师培训后，还要再经过1年-4年的专科医师培训，完成培训后才可参加专科医师资格考试^[3]^，中国也正在准备开始类似的“3+X”专科医师培训。这属于专科医师培训的内容，本文不做进一步的讨论。另外，临床研究生培养与住院医生规培并轨对研究生教育体系将造成一定程度的冲击，本文也不做进一步的讨论。

## 4 管理机构

与中国政府主导，政策和管理的主体是国家卫计委及其下属各级行政部门的管理机构设置不同，美国的住院医规培由毕业后医学教育认证委员会（Accreditation Council for Graduate Medical Education, ACGME）负责。ACGME为私立、非盈利性机构，由以下成员机构组成，包括：美国专业医生执照审批委员会（American Board of Medical Specialties）、美国医院协会（American Hospital Association）、美国医学会（American Medical Association）、美国医师学院协会（Association of American Medical College）和医学专业学会委员会，其董事会成员还包括两名住院医师和3名大众代表，以及一名联邦卫生部代表。其任务是保证住院医师培训的环境和质量，使其培训结束后能独立标准化行医，改善整体医疗水平。毕业后医学教育评审委员会是具体负责培训规范和认证培训机构，美国医学会配合制定详细的医生培训计划，美国医学专业委员会负责考核和颁发合格证书。在美国，每个培训单位必须符合上述机构的认证和监督。由其构成可以看出，ACGME更像是一个学术机构而不是行政机构。一方面其专家属性最大程度上避免了决策的盲目性，另一方面其非盈利属性在最大程度上减少了金钱交易。政府主导还是行业主导，一直是一个有争议的问题，暂且把美国模式放在一边，“简政放权”在我国也已经是一个老生常谈的问题。本届政府也一直把“简政放权”作为全面深化改革的“先手棋”和转变政府职能的“当头炮”，推动政府管理创新。在《国务院关于印发2015年推进简政放权放管结合转变政府职能工作方案的通知》中明确指出，“简政放权、放管结合和转变政府职能的任务更加紧迫、更加艰巨”。并着重强调要“深入推进职业资格改革，进一步清理和取消职业资格许可认定。制定行业组织承接水平评价类职业资格具体认定工作管理办法，推进水平评价类职业资格具体认定工作由行业协会等组织承担^[4]^。由此可以看出，在国家层面，还是希望行业协会等非政府组织来承担更多的社会责任，这是现代化国家发展的必然。目前在中国的执业医规培中，作为行业协会的中国医师协会和作为医学学术组织的中国医学会却完全处于从属地位。这一现状既与我们所借鉴的西方先进国家的经验相矛盾，也不符合我国的相关国策。规培主导权的归属问题，是住院医规培体系设计中的顶层设计，决定者规培的走向和最终的成败。或许中国可以借鉴美国的经验，由医学会、医师协会和医院管理协会等组织共同组建非官方的住院医师规范化培训的管理和组织机构，由卫计委派驻观察员，同时纳入住院医师和公众代表。这样的机构设置，可以最大程度上避免了政府部门管理效率低下、专业性缺乏的弊病，也符合国务院的相关文件精神。

## 5 质量控制

规培质量控制主要考虑于两个方面，一个方面是参加培训的住院医生，另一个方面是培训基地。

### 5.1 住院医生的筛选和考核

在美国，医生无疑是高吸引力的职业，其门槛之高也令人咋舌。如前文所述，能够进入住院医师培训者，已经经过了层层筛选，这是保证住院医师质量的非常重要的一环。而中国目前规培的门槛相对较低，基本上医学院校的本科毕业生都能申请到培训基地参加规培。而医学教育的现状是一方面是合格的临床医生的数量不能满足需求，另一方面是虽然医学院校大量扩招而毕业生大量放弃从事医疗行业。对规培基地而言，由于目前的规培生来源各异、起点水平相差很大，而且其待遇和身份也不尽相同，这些都导致管理上的困难。在3年的时间里让所有的住院医生进入统一设计的培训体系，达到住院医生培训的同质化，面临很大的困难。在目前情况下，即便建立严格的住院医师规培招录制度，也没有实际意义。这种状况并不是单纯通过完善规培制度能改变的，需要完善上游的政策和制度。

美国住院医师的工作强度非常大，曾经实行24小时住院制，外科住院医师每周通常工作110小时。1989年，为保障住院医师的休息时间，纽约州卫生部门在美国率先开始执行严格的规定限制住院医师的工作时间，即“405规则”。之后，ACGME提出了与“405规则”非常近似的规定，并于2003年7月7日起，在全国所有的教学医院开始执行。规定提出，住院医师一周工作不得超过80小时，而且值班之后必须休息10小时。与美国的情况不同，中国参加规培的住院医生中的大部分是在落实了工作单位之后再进入培训基地培训，缺乏竞争的压力，在积极性和主动性上是不足的。培训基地也不太可能对其有过于严格的要求。

美国医学研究中心（Institute of Medicine, IOM）和ACGME相继提出了住院医师胜任所应具备的核心能力。美国对所有专业的住院医师有一套非常完整和成熟的标准化考核体系，要求住院医师培训后掌握6项核心职业能力：临床能力（Patient Care and Procedural Skills）、医学知识（Medical Knowledge）、从临床实践中学习和自我提升的能力（Practice-based Learning and Improvement）、人际沟通和交流技能（Interpersonal and Communication Skills）、专业素质（Professionalism）和利用体系内资源的能力（Systems-based Practice）。国内对于住院医生应该拥有哪些技能和知识、应该具备何种素质和能力仍然没有统一的标准，缺乏系统性的规范。众多医院仍将住院医师培训的最后考核仍局限在临床技能和医学知识方面，在一定程度上错误指引了培训的方向。由于对住院医师培训目标在尺度把握和理解上存在差异，因此考核过程的规范性和客观性很难保证。人际沟通和交流技能的培训和考核在中国目前特殊的医疗环境下非常重要，却一直难以实施，不是因为重视程度不够，而是因为我国缺乏相应的方法和手段。在美国，ACGME和美国专科医师委员会（American Board of Medical Specialties, ABMS）于2000年9月共同公布了是围绕ACGME的六项核心能力制订的《毕业后医学教育受训者能力评估方法工具箱》。对每项能力都规定了相应的培训要求，而且对每项能力要求都提供了数种评估方法，并将方法分为三个等级，供各专科医师协会根据培训内容和阶段，有针对性地选择。对受训者的能力进行统一、全面的考核和评价^[5]^，对我国的住院医师规范化培训的考核工作极具借鉴作用。

### 5.2 培训机构的评定和审核

在美国，对承担住院医师培训的医院、医院内各专科及亚专科培训基地及所有的培训项目，ACGME均建立了统一审核标准，并负责全美住院医师培训基地的评估和审批及各专业中心课程的制定。培训机构资格认证类型与标准包括3种：培训住院医师机构要求、机构内各专科及专科培训基地的要求以及所有培训项目的统一要求^[6, 7]^。ACGME要求培养机构必须满足以下要求：①有书面材料向负责的机构申明；②有规范的管理系统；③有指定的机构官员；④提供足够的资源。美国医学教育联络委员会（Liaison Committee on Medical Education, LCME）负责对申请成为培训基地的医学院校附属医院或医学中心进行审核^[8]^。对于培训基地，ACGME要求其一定要附属于医学院，需建立培训基地委员会，明确轮转科室及完成培训内容的条件。

ACGME对于培训基地的认证标准涵盖病人数量、病种、培训教师数量和资格要求等方面。以急诊住院医师培训机构为例，ACGME要求该项目每年须至少有6名住院医师，其主要培训基地住院医与教师的比例为3:1，并应熟练掌握每一时期的培训目的和标准；年急诊量至少在3万人次以上，病种齐全，危重病或重伤的比例要至少占3%或1, 200人次以上，有各年龄段和不同性别的患者，并有强大的各专科会诊体系。ACGME对培训基地的相关管理人员和教师也提出了明确的要求。以急诊住院医师培训项目为例，基地主任要有3年以上教学经验，临床工作每周不超过20小时或每年960个小时。核心教师在参加临床教学的同时，要有足够的时间参与住院医师的培训，其平均临床工作不能超过每周28小时或每年1, 344小时。ACGME对外科住院医师培训期间主刀的手术量和具体操作也有明确的要求，如外科住院医师要求主刀手术（完成50%以上的手术过程）达到500例，每种手术也都要达到一定数量。在总住院医师期间至少主刀手术150例。ACGME每5年考察培训机构，不合格者将会被吊销培训能力，不能继续招收住院医生。在美国的住院医师培训基地或项目审批时，医院的相关科室必须向ACGME做出书面保证，确保住院医师在科室轮转培训过程中能充分享受到其指定的各种培训机会，包括各种操作。美国各专业学会组织每年一次全国住院医生培训考试（In Service Exam），一方面是客观检验住院医生的医学知识能力，另一方面也是考核培训机构的教育成果。培训教师大都积极参与住院医师的培训，会把住院医师需要掌握的各种知识、技能毫无保留地传授给他们。这一方面是因为住院医师培训的效果直接影响到基地和医院的声誉、培训基地的寿命；另一方面，完成住院医师培训内容也是教师的主要职责之一，若是教学医院的教师，其参与住院医师培训还与职称评定息息相关。

我国住院医师培训的最高管理机构是国家卫生计生委毕业后医学教育委员会，省级及以上卫生计生行政部门对住院医师培训基地进行认证。原则上培训基地设在三级甲等医院或以符合条件的三级医院和二级甲等医院作为补充。全科医生规范化培养基地除临床基地外还应当包括基层医疗卫生机构和专业公共卫生机构^[9]^。目前只对住院医师培训基地的软硬件设施进行考核，未对住院医师培训基地的承载能力做出相应的限制。这就很难保证基地能够提供足够的培训条件，更无法保证规培住院医生的学习和锻炼机会。为保证规培的质量，有必要加强对培训基地的认证和定期评估，并形成培训基地的淘汰机制，对评估不合格的培训基地进行警告直至淘汰。基地住院医担负了大量的值班、病历书写等繁杂的工作，而基地医院并不担负其薪酬，所以各培训基地从其单位利益考虑，不会轻易放弃其基地资格。而且，如果失去基地资格，其研究生培养也会受到很大的影响。另外，其声誉和学术地位也会受到损害。所以，各基地医院必然会最大限度的配合基地的认证和考核工作。

在美国，每个住院医生在结束每个轮转后都会受到以能力考查为主的全方位评定，包括出科考试以及带教老师与住院医生的双向评估。除培训教师对住院医师的每一个轮转做出评价外，美国住院医师有责任对培训基地主任、教师，及整个培训基地做出评价，这些资料均会在培训基地办公室备案，以备ACGME检查。培训基地的主任每年会与住院医师进行至少2次的一对一交流沟通。比如，该住院医师在培训过程中存在什么问题，是否有了改善，每个教师的评价如何等。同样，他也会查看住院医师对教师的评语，查看有没有哪位教师在培训过程中存在问题。倘若住院医师对带教教师不满，他可以举报该名带教教师。住院医师有权审看所有在他（她）的档案中的材料。不仅如此，在对住院医师培训基地每2-5年的重新评审过程中，ACGME会派人到医院驻点1天-2天。被派人员将与该医院的住院医师、带教教师、基地主任，及其他辅助人员进行谈话，检查培训人员档案，倘若发现有任何证实对此住院医师培训基地发展不利的现象或事件，ACGME将展开全面的调查并根据调查结果做出最终裁决，或待定以观后效，或者撤销基地资格，或者通过。

## 6 规培的薪酬

美国住院医生培训的费用是由国家联邦医疗保险和联邦医疗辅助计划服务中心（Centers for Medicare and Medicaid Services, CMS）支出。每年CMS投入的培训费用使用于直接和间接住院医生教育领域已经超过100亿美元，付于培训机构后大致每一住院医生10万美元。美国住院医生的福利待遇基本是全国统一。第一年住院医生的工资平均在5万美元左右，生活水准较高的大城市会稍高，比如在纽约第一年可以到5.5万，个别医院到达6万美元。随着住院医生年制升高每年工资有一定涨幅，第三年平均工资5.5万美元，根据美国司法部的数据，2014年伊利诺伊州的个人中位收入税前为48, 232美元，所以美国住院医师培训期间的工资水平以略高于当地的平均个人收入，在美国属于中等收入水平，能基本满足一个三口之家每月的生活开支。

中国的规培住院医生的薪酬主要三个不同来源，收入范围变动大概在2, 800元/月-5, 400元/月。以上海为例，人事关系所在地的市卫生和计划生育委员会负担规培生的基本工资，基本是1, 300元/月-1, 900元/月；所在规培单位，也就是各个作为规培基地的医院，负担规培生的保险、津贴及奖金等，大约是1, 500元/月；轮转科室发放的补助变动很大，是0元/月-2, 000元/月（以上为2013年-2014年的数据）。北京参加规培的住院医师的薪酬低于上海的水平，尤其是参加住院医师培训的研究生和没有工作单位的住院医生的薪酬更低，仅在2, 000元左右。其收入普遍处于一种勉强支付平时生活开销的窘境。根据北京市人力资源和社会保障局、北京市统计局的数据显示，2013年北京全市职工平均工资为69, 521元，月平均工资为5, 793元。深圳规培住院医生的薪酬较高，根据《医学界》的报道，目前深圳市规培生的生活补助标准是本科6, 000元/月、硕士7, 000元/月、博士8, 500元/月。除此之外不再有值班或加班的奖金或补贴，扣除社保（无住房公积金）和税后，本科每月约5, 500元，勉强可以维持在深圳的生活支出。而在实际操作中，住院医师的最低补助也没有保障，在部分地区和医院还出现了克扣规培医生补贴的情况。北京部分医院已经注意到规培住院医生的收入问题，并在医院层面给予一定的补助，如北京医院以医院的平均奖金（目前为5, 000元-6, 000元）为基数向规培住院医生发放奖金，第一年50%，第二年80%，第三年100%。总之，目前中国规培住院医生的薪酬极不合理，规培住院医师在很大程度上被视为书写病历、换药和手术拉钩的廉价劳动力。合理的薪酬不仅是社会公平性的要求，也是保证住院医生不为基本生活焦虑，全身心投入工作学习的基本条件，更关系到住院医生职业尊严和荣誉感的培养。为改变现状，首先是国家财政必须担负一定的责任，在一定时期内可以采取强制基地进行补贴、银行提供无息贷款等方式来缓解目前的困难。

美国住院医生培养的经济学逻辑是通过保证医生的高薪、高社会地位来保持其对社会精英的吸引力，使得成绩优异、素质超群的理工科大学毕业生有强烈的意愿突破重重考核进入医学院，并付出超常的努力以获得住院医生培训的机会。在住院医生培训阶段，由非政府的医学专业机构对住院医生和培训基地两个方面进行严格的考核，从而保证培训的高标准和高质量。医生的高水平，符合国民对生命和健康的重视和珍惜。如此，高水平的医疗体系提供高层次的健康保障、医生高付出获得高薪酬，非政府的专业管理提升了效率和公平。相比而言，中国医学院校招生的分数线在相对下降，住院医师规培门槛很低，而在岗医师在数量上也并不能满足社会需求，甚至出现儿科、急诊科等某些专业科室的医生荒，主管部门被迫饮鸩止渴，通过降低入职要求来缓解矛盾。在此大背景下，不努力改善规培住院医生的生存环境，片面提升规培住院医生的标准和要求，在内部继续恶化住院医师的生存环境，很可能适得其反，进一步降低行业的吸引力。

## 7 结语

美国住院医生培训已经实施很多年，有很多值得借鉴的合理性和科学性。中国的住院医生规培刚刚起步，不可能与其比肩。但是，必须对中国规培的走向进行深入的分析和充分的探讨，确保规培在正确的道路上不断发展。政府和国民对健康权和生命权的重视和尊重必然会不断加强，医生的素质和技能也必须随之不断的进步和提升，这就决定了规培的质量和要求只能不断地提高。基于对中美两国培训制度和体系的比较和分析，本文做出以下总结：①增加财政投入，尤其是国家层面的财政投入；②建立非政府的专业化管理机构；③加强规培质量管理，首先是加强对培训基地的管理和考核。
